# A Treatise on Human Physiology

**Published:** 1864-04

**Authors:** 


A Treatise on Human Physiology: Designed for the Use of Students and Practitioners of Medicine. By John C. Dalton, Jr., M.D., Prof. of Physiology and Microscopic Anatomy in the College of Physicians and Surgeons, New York; Member of the New York Academy of Medicine; of the New York Pathological Society; of the American Academy of Arts and Sciences, Boston, Mass.; and of the Biological Department of the Academy of Natural

Sciences, of Philadelphia. Third Edition, revised and enlarged. With Two Hundred and Seventy-three Illustrations.

The fact that a third edition of this work has been called for in so short a time, shows that it is already well known, and its merits appreciated by the profession.

It is one of the most concise and valuable works on human physiology in the English language. It is published in the best style of Blanchard & Lea, of Philadelphia; and for sale by W. B. Keen, 148 Lake Street, Chicago.

